# Electron Microscopic Radioautographic Study on Mitochondrial DNA Synthesis in Adrenal Cortical Cells of Developing and Aging Mice

**DOI:** 10.1100/tsw.2008.93

**Published:** 2008-07-13

**Authors:** Tetsuji Nagata

**Affiliations:** ^1^ Department of Anatomy and Cell Biology, Shinshu University School of Medicine, Matsumoto, 390-8621, Japan; ^2^ Department of Anatomy, Shinshu Institute of Alternative Medicine and Welfare, Nagano, 380-0816, Japan

**Keywords:** mitochondria, mouse adrenal cortex, EM radioautography, DNA synthesis

## Abstract

In order to study the aging changes of intramitochondrial DNA synthesis of mouse adrenal cortical cells, eight groups of developing mice, each consisting of three individuals (total 24), from fetal day 19 to postnatal newborn at days 1, 3, 9, 14, to adult at months 1, 2, and 6, were injected with ^3^H-thymidine, sacrificed 1 h later, and the adrenal tissues were fixed and processed for electron microscopic (EM) radioautography. On EM radioautograms obtained from each animal, the number of mitochondria and the mitochondrial labeling index labeled with ^3^H-thymidine showing DNA synthesis in each adrenal cortical cell, in three zones, were counted and the results in respective developing groups were compared. From the results, it was demonstrated that the numbers of mitochondria in the three zones, the zona glomerulosa, fasciculata, and reticularis, of mice at various ages increased from fetal day 19 to postnatal month 6 due to development and aging of animals, respectively, while the number of labeled mitochondria and the labeling index of intramitochondrial DNA syntheses incorporating ^3^H-thymidine increased from fetal day 19 to postnatal month 2, reaching the maxima, and decreased to month 6. It was shown that the activity of intramitochondrial DNA synthesis in the adrenal cortical cells in developing and aging mice changed due to aging.

### INTRODUCTION

Intramitochondrial nucleic acid syntheses, both DNA and RNA, in mammalian and avian cells were first demonstrated morphologically by the present author by means of electron microscopic (EM) radioautography, with accurate localization in primary cultured cells of the livers and kidneys of mice and chickens *in vitro*[1,2] and then in some other established cell lines, such as HeLa cells[3,4,5,6] or mitochondrial fractions prepared from *in vivo* cells[7,8,9]. They were later commonly found in various cells and tissues not only *in vitro* obtained from various organs *in vivo*[10,11,12,13,14], but also *in vivo* cells of various organs such as the salivary gland[15], liver[16,17,18,19,20,21,22,23,24,25,26,27,28,29], pancreas[30,31], trachea[32], lung[33], kidney[34], testis[35,36], uterus[37,38], adrenal gland[39,40,41], brain[42], and retina[43,44,45,46,47] of mice, rats, and chickens. The relationship between the intramitochondrial DNA synthesis and cell cycle was formerly studied and it was clarified that the intramitochondrial DNA synthesis was performed without nuclear involvement[3]. However, the relationship between the DNA synthesis in the adrenal cortical cells and the aging of individual animals has not yet been clarified. This paper deals with the relationship between the DNA synthesis and development in adrenal cortical cells of mice *in vivo* at various developmental stages from fetal day 19 to postnatal month 2 by means of EM radioautography as a part of serial studies on special cytochemistry[48] and radioautographology[49].

### MATERIALS AND METHODS

#### The Animals

The adrenal tissues were obtained from eight groups of developing and aging normal ddY strain mice, each consisting of three individuals (total 24), from fetal day 19 to postnatal newborn at days 1, 3, 9, 14, to adult at months 1, 2, and 6, each consisting of three littermates of both sexes. The embryonic age was based on observation of the vaginal plug of the female mice (vaginal plug = day 0). All the animals were housed under conventional conditions and bred with normal diet (mouse chow Clea EC2, Clea Co., Tokyo, Japan) with access to water *ad libitum* in our laboratory. They were administered with $^3$H-thymidine, DNA precursor, and the adrenal tissues were fixed and processed for EM radioautography. All the procedures used in this study concerning the animal experiments were in accordance with the guidelines of the animal research committee of Shinshu University School of Medicine, as well as the principles of laboratory animal care in NIH publication No. 86-23 (revised 1985).

#### Electron Microscopic Radioautography

All the animals were injected intraperitoneally with $^3$H-4-thymidine (Amersham, England, specific activity 877 GBq/m*M*) in saline, at 9 a.m., 1 h before sacrifice. The dosage of injections was 370 KBq/g body weight. The animals were perfused at 10 a.m., 1 h after the injection, via the left ventricles of the hearts with 0.1 *M* cacodylate-buffered 2.5% glutaraldehyde under Nembutal (Abbott Laboratories, Chicago, IL, USA) anesthesia. The right adrenal glands were taken out, excised, and three small pieces of the adrenal tissues (1 × 1 × 1 mm) were immersed in the same fixative at 4˚C for 1 h, followed by postfixation in 1% osmium tetroxide in the same buffer at 4˚C for 1 h, dehydrated in graded series of ethanol and acetone, and embedded in epoxy resin Epok 812 (Oken, Tokyo, Japan).

For EM radioautography, semithin sections at 0.2 µm thickness, thicker than conventional ultrathin sections in order to shorten the exposure time, were cut in sequence on a Porter-Blum MT-2B ultramicrotome (Dupont-Sorvall, Newtown, MA, USA) using glass knives. The sections were collected on collodion-coated copper grid meshes (VECO, Eerbeek, The Netherlands), coated with Konica NR-H2 radioautographic emulsion (Konica, Tokyo, Japan) by a wire-loop method[30,48,49,50,51]. They were stored in dark boxes containing silica gel (desiccant) at 4˚C for exposure. After the exposure for 10 months, the specimens were processed for development in freshly prepared gold latensification solution for 30 sec at 16˚C and then in fresh phenidon developer for 1 min at 16˚C in a water bath, rinsed in distilled water, and dried in an oven at 37˚C overnight, stained with lead citrate solution for 3 min, coated with carbon for electron microscopy. The EM radioautograms were examined in a JEOL JEM-4000EX electron microscope (JEOL, Tokyo, Japan) at accelerating voltages of 400 kV for observing thick specimens.

### Quantitative Analysis of Electron Micrographs

For quantitative analysis of electron micrographs, 20 EM radioautograms showing cross-sections of adrenal cortical cells from each group, based on the EM photographs taken after observation on at least 100 adrenal cortical cells from respective animals, and at least 10 cells from respective zones, i.e., glomerulosa, fasciculata, and reticularis, were analyzed to calculate the total number of mitochondria in each adrenal cortical cell in respective zones, i.e., glomerulosa, fasciculata, and reticularis, and the number of labeled mitochondria covered with silver grains by visual grain counting.

On the other hand, the number of silver grains in the same area size as a mitochondrion outside cells was also calculated in respective specimens as background fog, which resulted in less than one silver grain (0.03/mitochondrial area), almost zero. Therefore, the grain count in each specimen was not corrected with background fog. From all the data thus obtained, the averages and standard deviations in respective aging groups were computed with a personal computer (Macintosh type 8100/100, Apple Computer, Tokyo, Japan). The data were stochastically analyzed using variance and Student's t-test. The differences were considered to be significant at $p < 0.01$.

## RESULTS

### Morphological Observations

The adrenal cortical tissues obtained from ddY strain mice at various ages from embryo day 19 to postnatal day 30, consisted of three layers, zona glomerulosa, zona fasciculata, and zona reticularis, developing gradually. At embryonic day 19 and postnatal day 1, the adrenal cortical cells were composed mainly of polygonal cells, while the specific orientation of the three layers, zona glomerulosa (Fig. 1), zona fasciculata (Fig. 2), and zona reticularis (Fig. 3), was not yet well established. At postnatal day 3, the orientation of the three layers, zona glomerulosa (Fig. 4), zona fasciculata (Fig. 5), and zona reticularis (Fig. 6), became evident. At postnatal days 9 and 14, the specific structure of the three layers was completely formed and the arrangements of the cells in respective layers became typical, especially at day 14 (Figs. 7, 8, 9). Observing the ultrastructure of the adrenal cortical cells, cell organelles including mitochondria were not so well developed at perinatal and early postnatal stages from embryonic day 19 (Figs. 1, 2, 3) to postnatal day 3 (Figs. 4, 5, 6). However, these cell organelles, mitochondria, endoplasmic reticula, Golgi apparatus, appeared well developed from the juvenile stage at postnatal day 14 (Fig. 7, 8, 9) to the adult stages at postnatal months 1 (Figs. 10, 11, 12), 2 (Figs. 13, 14, 15), and 6 (Figs. 16, 17, 18). The zona glomerulosa was the thinnest layer found at the outer zone, covered by the capsule, and consisted of closely packed groups of columnar or pyramidal cells forming arcades of cell columns. The cells contained many spherical mitochondria and well-developed smooth-surfaced endoplasmic reticula, but a compact Golgi apparatus in day 14 animals (Fig. 7). The zona fasciculata was the thickest layer, consisted of polygonal cells that were larger than the glomerulosa cells, and arranged in long cords disposed radially to the medulla containing many lipid droplets (Fig. 8). At postnatal month 1, the specific structure of the three layers (Figs. 10, 11, 12) was completely developed and the arrangements of the cells in respective layers became typical as adult tissues. Observing the ultrastructure of the adrenal cortical cells at the juvenile and adult stages, cell organelles including mitochondria were well developed from postnatal day 14 to months 1 (Figs. 10, 11, 12), 2 (Figs. 13, 14, 15), and 6 (Figs. 16, 17, 18). The mitochondria in the zona fascilulata were less numerous and were more variable in size and shape than those of the glomerulosa cells, while the smooth-surfaced endoplasmic reticula were more developed and the Golgi apparatus was larger than the glomerulosa. In the zona reticularis (Figs. 9, 12, 15, 18), the parallel arrangement of cell cords was anastomosed, showing networks continued to the medullar cells. The mitochondria were less numerous and were more variable in size and shape than those of the glomerulosa cells like the fasciculata cells, and the smooth-surfaced endoplasmic reticula were developed and the Golgi apparatus was large like the fasciculata

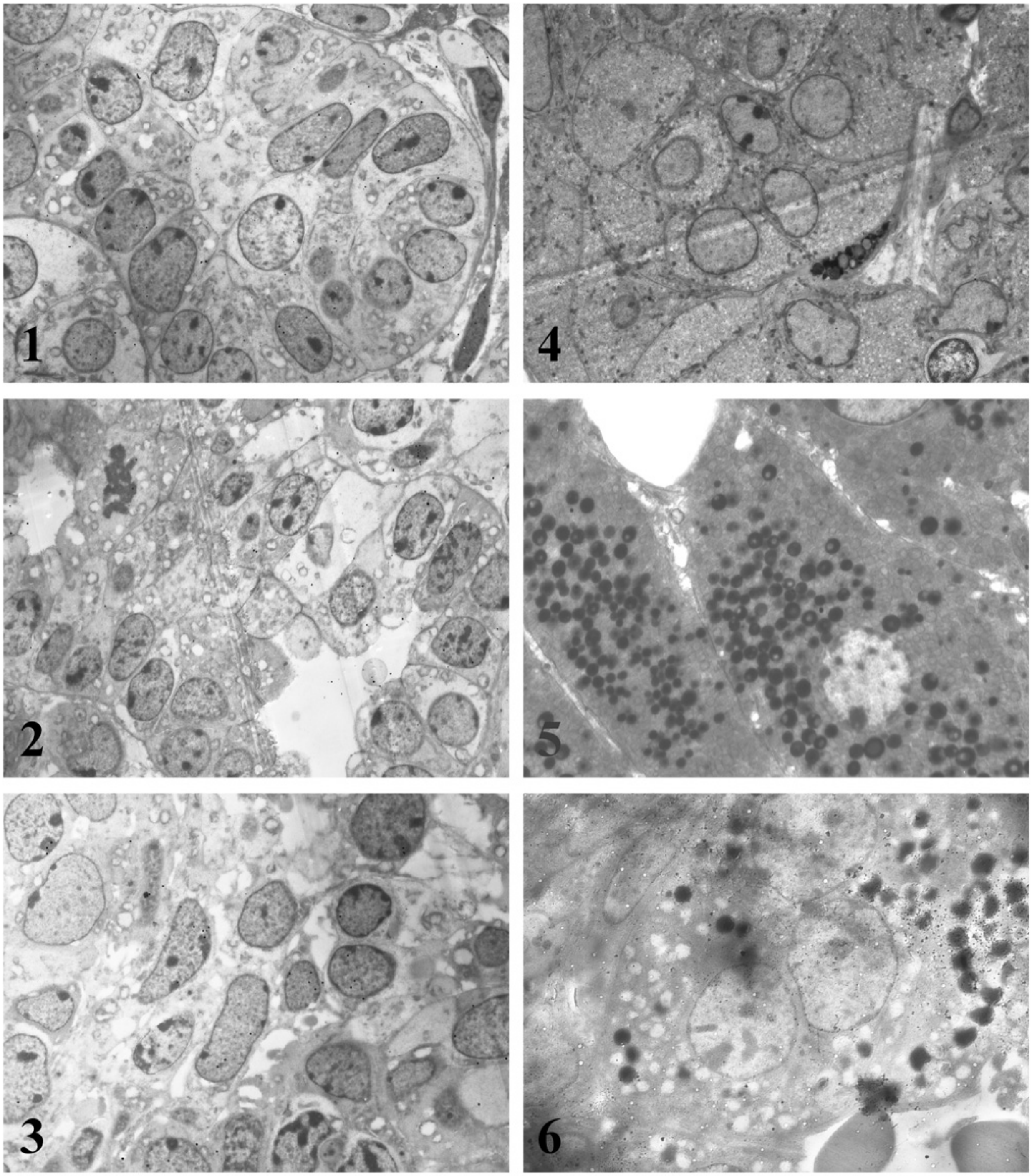

**FIGURES 1–.3** EM radioautograms of the zona glomerulosa **(1)**, zona fasciculata **(2)**, and zona reticularis **(3)** of a mouse embryo at fetal day 19, labeled with $^{3}$H-thymidine showing DNA synthesis (several silver grains) in the nucleus as well as in a few mitochondria. × 3,000.

**FIGURES 4–6.** EM radioautograms of the zona glomerulosa **(4)**, zona fasciculata **(5)**, and zona reticularis **(6)** of a newborn mouse aged at postnatal day 3, labeled with $^{3}$H-thymidine showing DNA synthesis (a few silver grains) in the nucleus (upper right) as well as in several mitochondria of the three adrenal cortical cells. × 3,000.

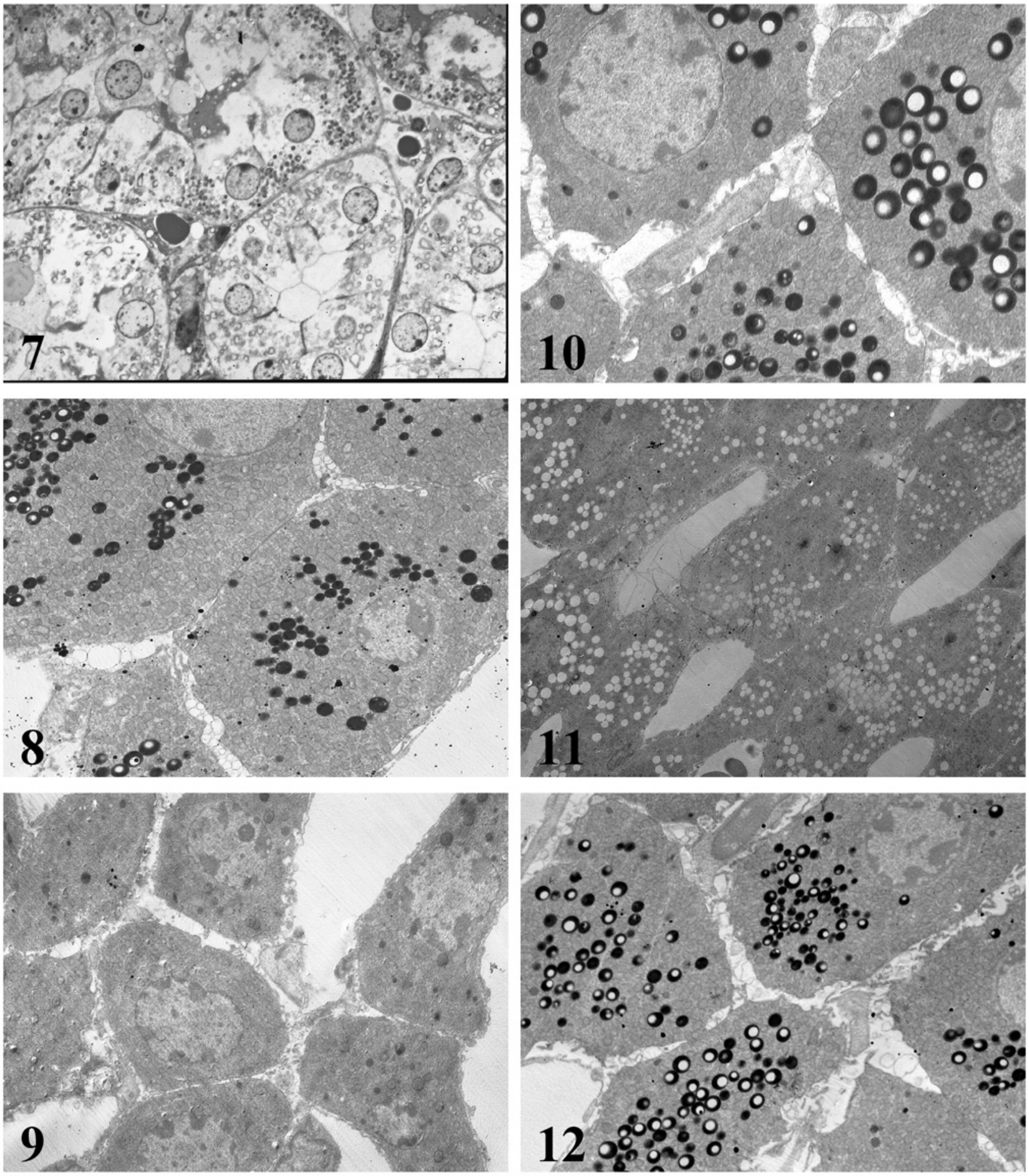

**FIGURES 7–9.** EM radioautograms of a juvenile mouse aged at postnatal day 14, labeled with $^3$H-thymidine showing: **(7)** DNA synthesis (several silver grains) in several nuclei as well as in several mitochondria in several adrenal cortical cells, zona glomerulosa, × 1,500; **(8)** DNA synthesis (several silver grains) in the nucleus (upper) as well as in several mitochondria in the two adrenal cortical cells, zona fasciculata, × 3,000; **(9)** DNA synthesis in several mitochondria (with silver grains), but not in the nuclei (no silver grain), zona reticularis, × 3,000.

**FIGURES 10–12.** EM radioautograms of a young adult mouse aged at postnatal month 1, labeled with $^3$H-thymidine showing: **(10)** DNA synthesis (several silver grains) in several nuclei as well as in several mitochondria in several adrenal cortical cells, zona glomerulosa, × 1,500; **(11)** DNA synthesis (several silver grains) in the nucleus (upper) as well as in several mitochondria in the two adrenal cortical cells, zona fasciculata, × 3,000; **(12)** DNA synthesis in several mitochondria (with silver grains), but not in the nuclei (no silver grain), zona reticularis, × 3,000.

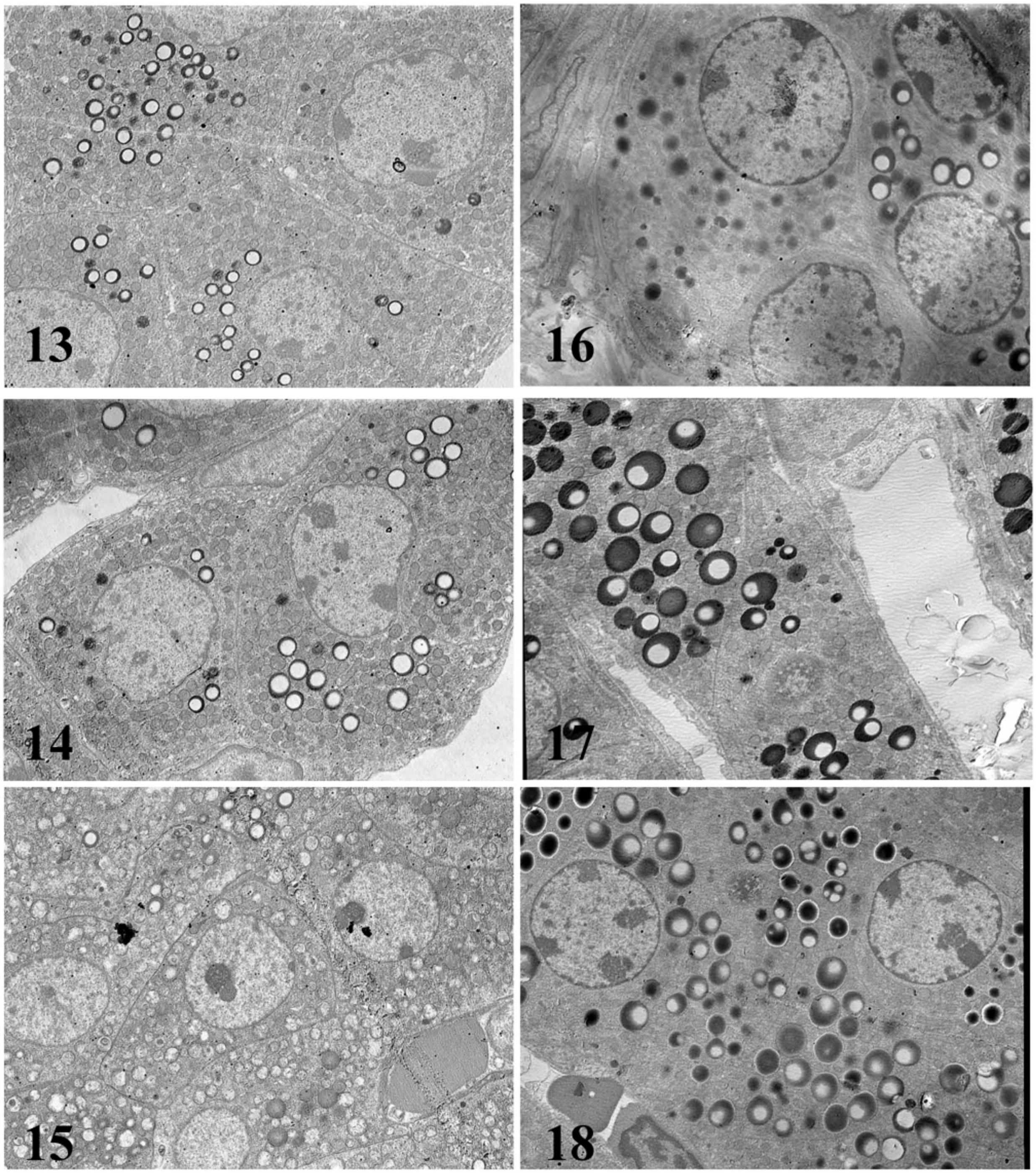

**FIGURES 13–15.** EM radioautograms of a mature adult mouse aged at postnatal month 2, labeled with $^3$H-thymidine showing: **(13)** DNA synthesis (several silver grains) in several nuclei as well as in several mitochondria in several adrenal cortical cells, zona glomerulosa, × 1,500; **(14)** DNA synthesis (several silver grains) in the nucleus (upper) as well as in several mitochondria in the two adrenal cortical cells, zona fasciculata, × 3,000; **(15)** DNA synthesis in several mitochondria (with silver grains), but not in the nuclei (no silver grain), zona reticularis, × 3,000.

**FIGURES 16–18.** EM radioautograms of an old adult mouse aged at postnatal month 6, labeled with $^3$H-thymidine showing: **(16)** DNA synthesis (several silver grains) in several nuclei as well as in several mitochondria in several adrenal cortical cells, zona glomerulosa, × 1,500; **(17)** DNA synthesis (several silver grains) in the nucleus (upper) as well as in several mitochondria in the two adrenal cortical cells, zona fasciculata, × 3,000; **(18)** DNA synthesis in several mitochondria (with silver grains), but not in the nuclei (no silver grain), zona reticularis, × 3,000.

cells. Thus, the structure of the adrenal cortical cells showed changes due to development and aging at respective developmental stages.

### Radioautographic Observations

Observing EM radioautograms, the silver grains were found over the nuclei of some adrenal cortical cells labeled with $^3$H-thymidine, demonstrating DNA synthesis mainly in perinatal stages at embryonic day 19 (Figs.1, 2, 3), postnatal days 1 and 3 (Figs. 4, 5, 6), while less at days 9 and 14 (Figs. 7, 8, 9) and adults at months 1 (Figs. 10, 11, 12), 2 (Figs. 13, 14, 15), and 6 (Figs. 16, 17, 18).

However, those labeled cells were found in all the three layers, the zona glomerulosa (Figs. 1, 4, 7, 10, 13, 16), the zona fasciculata (Figs. 2, 5, 8, 11, 14, 17), and the zona reticularis (Figs. 3, 6, 9, 12, 15, 18), at respective aging stages. In the labeled adrenal cortical cells in the three layers, the silver grains were mainly localized over the euchromatin of the nuclei and only a few or several silver grains were found over the mitochondria of these cells. To the contrary, most adrenal cortical cells were not labeled with any silver grains in their nuclei nor cytoplasm, showing no DNA synthesis even after labeling with $^3$H-thymidine. Among many unlabeled adrenal cortical cells, most cells in the three layers were observed to be labeled with several silver grains over their mitochondria due to the incorporation of $^3$H-thymidine, especially at the perinatal stages from embryonic day 19 (Figs. 1, 2, 3) to postnatal days 1, 3 (Figs. 4, 5, 6), 9, and 14 (Figs. 7, 8, 9). The localizations of silver grains over the mitochondria were mainly on the mitochondrial matrices when observed by high-power magnification (Fig. 15).

### Quantitative Analysis

#### Number of Mitochondria per Cell

Preliminary quantitative analysis on the number of mitochondria in 10 adrenal cortical cells whose nuclei were labeled with silver grains and another 10 cells whose nuclei were not labeled in each aging group revealed that there was no significant difference between the number of mitochondria and the labeling indices ($p < 0.01$). Thus, the number of mitochondria and the labeling indices were calculated regardless of whether their nuclei were labeled or not. The results obtained from the number of mitochondria in adrenal cortical cells in the three layers of respective animals in eight aging groups at perinatal stages, prenatal embryo day 19, and postnatal days 1, 3, 9, and 14, showed a gradual increase from the prenatal day 19 (glomerulosa 12.5, fasciculata 14.9, reticularis 15.2/cell) to postnatal day 14 (glomerulosa 37.8, fasciculata 37.3, reticularis 40.9/cell), and to adult stages at postnatal months 1 (glomerulosa 42.4, fasciculata 42.0, reticularis 42.9/cell), 2 (glomerulosa 64.2, fasciculata 64.0, reticularis 68.2/cell), and 6 (glomerulosa 59.8, fasciculata 62.9, reticularis 62.9/cell), as shown in Fig. 19. The increase from embryo day 19 to postnatal month 6 was stochastically significant ($p < 0.01$).

#### Mitochondrial DNA Synthesis

The results of visual grain counting on the number of mitochondria labeled with silver grains obtained from 10 adrenal cortical cells in the three layers of each animal labeled with $^3$H-thymidine demonstrating DNA synthesis in eight aging groups at perinatal stages, prenatal embryo day 19, postnatal days 1, 3, 9, and 14, and months 1, 3, and 6, are plotted in Fig. 20. The results demonstrated that the numbers of labeled mitochondria with $^3$H-thymidine showing DNA synthesis per cell gradually increased from prenatal embryo day 19 (glomerulosa 0.3, fasciculata 0.5, reticularis 0.4/cell) to postnatal day 14 (glomerulosa 1.5, fasciculata 1.5, reticularis 1.6/cell), and months 1 (glomerulosa 1.8, fasciculata 1.8, reticularis 2.2/cell) and 2 (glomerulosa 5.4, fasciculata 5.0, reticularis 6.1/cell), reaching the maximum, then decreased to month 6 (glomerulosa 2.4, fasciculata 2.6, reticularis 2.7/cell).

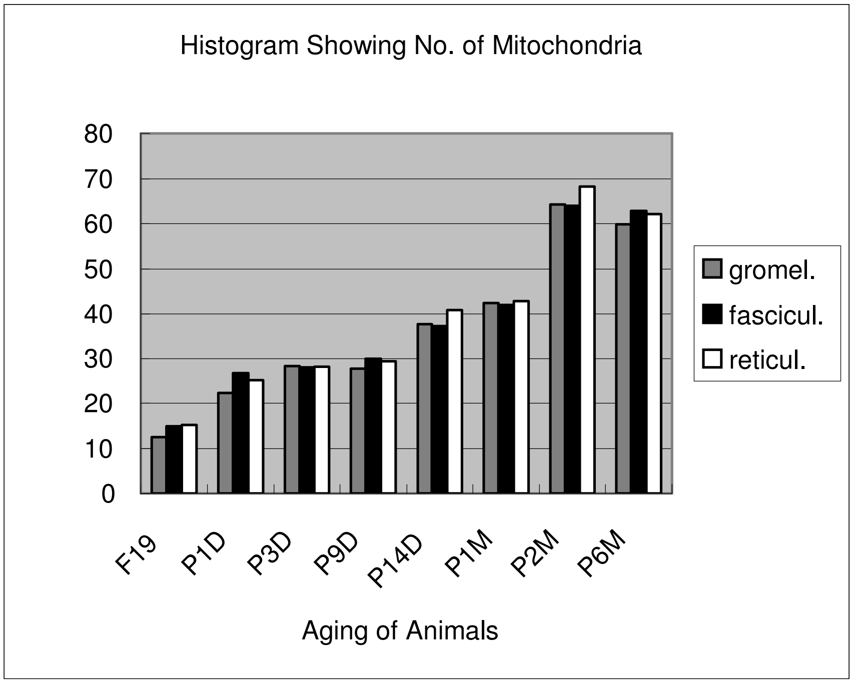

**FIGURE 19.** Histogram showing the number of mitochondria per each adrenal cortical cell in the three zones of respective animals in eight groups during the development and aging from fetal day 19 to postnatal month 6. The number of mitochondria per cell increased from perinatal stage to old stage at postnatal month 6.

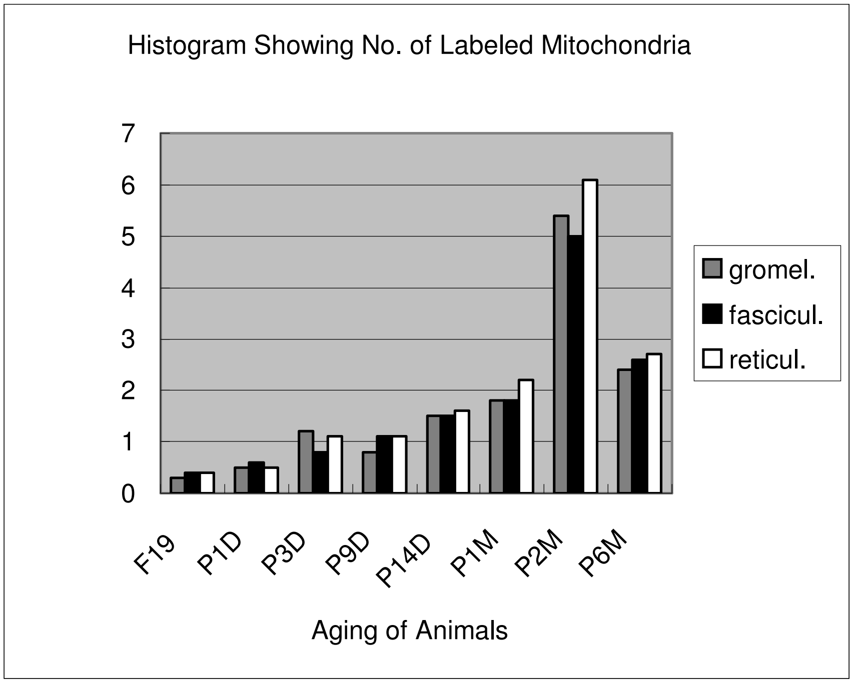

**FIGURE 20**. Histogram showing the number of labeled mitochondria per each adrenal cortical cell in the three zones of respective animals in eight groups during the development from fetal day 19 to postnatal month 6. The number of labeled mitochondria per cell increased from perinatal stage to adult stage at postnatal month 2, then decreased at month 6.

### *The Labeling Index*

On the other hand, the labeling indices in respective aging stages were calculated from the number of labeled mitochondria (Fig. 20) divided by the number of total mitochondria per cell (Fig. 19), which were plotted in Fig. 21, respectively.

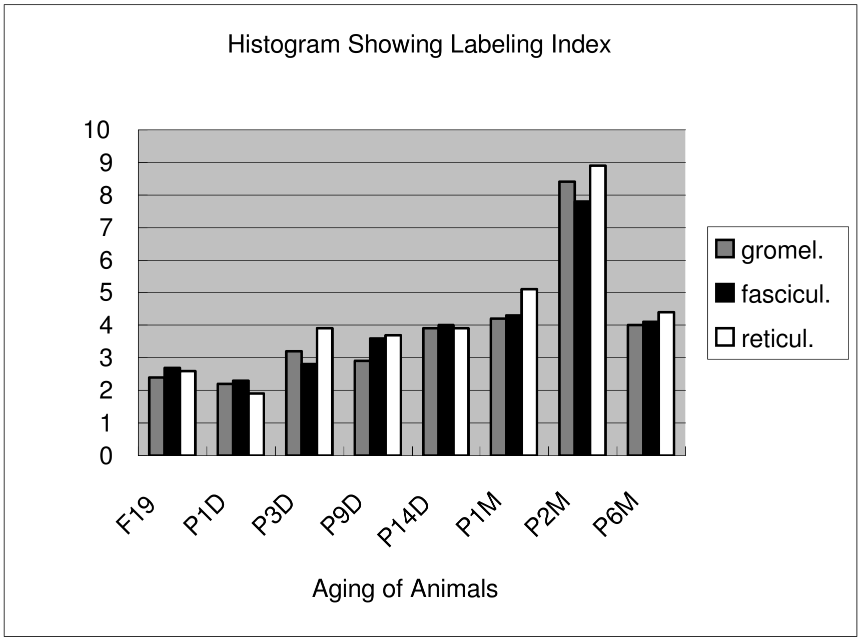

**FIGURE 21.** Histogram showing the labeling index of mitochondria per each adrenal cortical cell in the three zones of respective animals in eight groups during the development from fetal day 19 to postnatal month 6. The labeling index of mitochondria increased from perinatal stage to adult stage at postnatal month 2, then decreased at month 6.

The results showed that the labeling indices gradually increased from prenatal day 19 (glomerulosa 2.4, fasciculata 2.7, reticularis 2.6%) to postnatal juvenile stage at day 14 (glomerulosa 3.9, fasciculata 4.0, reticularis 3.9%) and to the adult stage at months 1 and 2 (glomerulosa 8.4, fasciculata 7.8, reticularis 8.9%), reaching the maximum, and decreased to month 6 (glomerulosa 4.0, fasciculata 4.1, reticularis 4.4%), as shown in Fig. 21.

### DISCUSSION

From the results obtained at present, it was shown that intramitochondrial DNA synthesis was observed in adrenal cortical cells in the three layers, zona glomerulosa, zona fasciculata, and zona reticularis, of developing and aging mice at various ages from prenatal embryos to postnatal newborn, young juvenile, and adult stages, and the number of mitochondria per cell showed increases due to aging, while the number of labeled mitochondria per cell and the labeling indices showed increases and decreases due to aging.

As for the macromolecular synthesis in various cells in various organs of experimental animals observed by light and electron microscopic radioautography, it is well known that the silver grains due to radiolabeled $^{3}$H-thymidine demonstrate DNA synthesis[1,2,3,48,49]. The previous results obtained from the studies on the adrenal cortical cells of aging mice by light microscopic radioautography revealed that silver grains indicating DNA synthesis incorporating $^{3}$H-thymidine were observed over the nuclei of some adrenal cortical cells at perinatal stages from postnatal day 1 to 14[39,40]. However, they did not observe the intramitochondrial DNA synthesis. In the present study, the numbers of silver grains showing nuclear DNA synthesis, as expressed by grain counting, did not give any significant difference between the cells in the three layers in the same aging groups. These results indicated that the amount of DNA synthesized in one nucleus was almost the same as in any other cells independent of whether the nucleus belonged to any layers of the adrenal cortex. However, these differences between the three layers at respective aging groups were not stochastically significant ($p < 0.01$). These results indicated that the DNA synthetic activity in the nuclei of three layers of the adrenal cortex did not show any difference. To the contrary, the labeling indices between the aging groups increased gradually from prenatal embryo day 19, to postnatal days 1, 3, 9, and 14, to postnatal months 1 and 2, then decreased to month 6. This decrease was stochastically significant ($p < 0.01$).

On the other hand, the radioautograms in the present study, showing incorporations of $^{3}$H-thymidine into mitochondria, indicating mitochondrial DNA synthesis, resulted in silver grain localization over the mitochondria independently from the nuclei whether the nuclei were labeled with silver grains or not in almost all the cells in the three layers of the adrenal cortical cells from prenatal embryo day 19 to postnatal days 1, 3, 9, and 14, to postnatal months 1, 2, and 6, during development and aging. The numbers of labeled mitochondria showing DNA synthesis as well as the labeling indices increased from perinatal embryonic day (glomerulosa 2.4, fasciculata 2.7, reticularis 2.6%) to postnatal newborn and juvenile stages at day 14 (glomerulosa 3.9, fasciculata 4.0, reticularis 3.9%) to the adult stages at months 1 and 2 (glomerulosa 8.4, fasciculata 7.8, reticularis 8.9%), reaching the maxima, but decreased at month 6.

With regards to DNA in mitochondria in animal cells or plastids in plant cells, many studies have reported on various cells of various plants and animals since the 1960s[52,53,54,55]. Most of these authors observed DNA fibrils in mitochondria that were histochemically extracted by DN'ase. EM observation of the DNA molecules isolated from the mitochondria revealed that they were circular in shape, with a circumference of 5–6 µm[56]. It was calculated that such a single molecule had a molecular weight of about 107 Da[57]. Mitochondria of various cells also contained a DNA polymerase, which was supposed to function in the replication of the mitochondrial DNA[58]. On the other hand, the incorporation of $^{3}$H-thymidine into mitochondria demonstrating DNA synthesis was observed by means of EM radioautography in lower organisms such as slime mold[59,60], tetrahymena[61], or chicken fibroblasts in tissue culture under abnormal conditions[62]. However, these authors used old-fashioned developers consisting of methol and hydroquinone (MQ-developer), which produced coarse, spiral silver grains resulting in inaccurate localization over cell organelles when observed by electron microscopy. All of these authors showed photographs of electron radioautographs with large, spiral-formed silver grains (2–3 µm in diameter) localizing not only over the mitochondria, but also outside the mitochondria. In order to obtain smaller silver grains, we first used elon-ascorbic acid developer after gold latensification[1,2,3,4,5,6], which produced smaller, comma-shaped silver grains (0.4–0.8 µm in diameter), then later we used phenidon developer after gold latensification, producing smaller, dot-like silver grains (0.2–0.4 µm in diameter), localizing only inside the mitochondria showing ultrahigh resolution of radioautograms[30,48,49,50,51]. These papers were the first to demonstrate intramitochondrial DNA synthesis incorporating $^{3}$H-thymidine with accurate intramitochondrial localization in avian and mammalian cells. With regards to the resolution of EM radioautography, on the other hand, many authors discussed the sizes of silver grains under various conditions and calculated various values of resolutions[4,5,63,64,65]. Those authors who used the MQ developers maintained the resolution to be 100–160 nm[63,64], while those authors who used the elon-ascorbic acid developer[4,5,65] calculated it to be 25–50 nm. When we used phenidon developer at 16˚C for 1 min after gold latensification, we could produce very fine, dot-shaped silver grains and obtained the resolution around 25 nm[30,48,49,50,51,66]. For the analysis of electron radioautographs, Salpeter et al.[63] proposed to use the half-distance and very complicated calculations through which respective coarse, spiral-shaped silver grains were judged to be attributable to the radioactive source in a certain territory within a resolution boundary circle. However, since we used phenidon developer after gold latensification to produce very fine, dot-shaped silver grains, we judged only the silver grains that were located in the mitochondria that were those very fine, dot-shaped ones to be attributable to the mitochondria without any problem as was formerly discussed[3,4,5,48,49,50,51].

Then we also demonstrated intramitochondrial DNA synthesis incorporating $^3$H-thymidine in some other established cell lines originated from human beings, such as HeLa cells[3,4,5,6], or mitochondrial fractions prepared from *in vivo* mammalian cells, such as rat and mouse[7,8,9]. It was later commonly found in various cells and tissues not only *in vitro* obtained from various organs *in vivo*, such as the cultured human HeLa cells[13,67], cultured rat sarcoma cells[12], and mouse liver and pancreas cells *in vitro*[11,14,31], but also *in vivo* cells obtained from various organs such as the salivary gland[15], liver[16,17,18,19,20,21,22,23,24,25,26,27,28,29], pancreas[30,31], trachea[32], lung[33], kidney[34], testis[35,36], uterus[37,38], adrenal gland[39,40,41], brain[42], and retina[43,44,45,46,47] of mice, rats, and chickens. Thus, it is clear that all the cells in various organs of various animals synthesize DNA not only in their nuclei, but also in their mitochondria.

The relationship between the intramitochondrial DNA synthesis and cell cycle was formerly studied in synchronized cells and it was clarified that the intramitochondrial DNA synthesis was performed without nuclear involvement[4]. However, the relationship between the DNA synthesis and the aging of individual animals and men has not yet been clarified except in a few papers recently published by Korr and associates on mouse brain[67,68,69,70]. They reported both nuclear DNA repair, measured as nuclear unscheduled DNA synthesis, and cytoplasmic DNA synthesis labeled with $^3$H-thymidine in several types of cells in brains, such as pyramidal cells, Purkinje cells, granular cells, glial cells, endothelial cells, ependymal cells, and epithelial cells as observed by light microscopic radioautography using paraffin sections. They observed silver grains over cytoplasm of these cells by light microscopy and maintained that it was reasonable to interpret this labeling as $^3$H-DNA outside the nuclei, which theoretically belonged to mitochondrial DNA without observing the mitochondria by electron microscopy. From the results, they concluded that distinct types of neuronal cells showed a decline of both unscheduled DNA and mitochondrial DNA syntheses with age; in contrast, that of other cell types, glial and endothelial cells, did not show such age-related changes without counting the number of mitochondria in respective cells or counting the labeling indices at respective aging stages. Thus, their results from the statistics obtained from the cytoplasmic grain counting seem to be not accurate without observing mitochondria directly. To the contrary, we studied DNA synthesis in the livers of aging mice[16,17,18,19,20,21,22, 23,24,25,26,27,28,29] and clearly demonstrated that the number of mitochondria in each hepatocyte, especially mononucleate hepatocytes, increased with the ages of animals from the perinatal stages to adult and senescent stages, while the number of labeled mitochondria and the labeling indices increased from the perinatal stages, reaching a maximum at postnatal day 14, then decreased. Our previous studies[22,23] also clarified that the DNA synthesis and cell proliferation by mitosis were the most active in the nuclei of mononucleate hepatocytes at the perinatal stages; in contrast, binucleate cells were less active at the perinatal stage, but the number of binucleate hepatocytes increased at senescent stages and the results suggest the possibility that the mitochondria in mononucleate hepatocytes synthesized their DNA by themselves, which peaked at postnatal day 14 in accordance with the proliferation of mononucleate hepatocytes, while binucleate hepatocytes increased after the perinatal stage and did not divide, but remained binucleate, keeping many mitochondria in their cytoplasm, which were more in number than mononucleate hepatocytes at the senescent stage.

Thus, our previous papers were the first to deal with the relationship between the DNA synthesis and aging in hepatocytes of mice *in vivo* at various ages by means of EM radioautography observing the small, dot-like silver grains, due to incorporations of $^3$H-thymidine, which exactly localized inside the mitochondria. The present results also revealed that an increase was observed by direct observation on mitochondria at EM level, and accurate mitochondrial number and labeling indices in adrenal cortical cells in eight groups of developing mice were obtained. There was a discrepancy between our results from the hepatocytes[22,23] as well as the adrenal cortical cells at present and the results from the several types of cells in the brains by Korr et al.[67,68,69,70]. The reason for this difference might be due to the difference between the cell types (hepatocytes or adrenal cells and the brain cells) or the difference between the observation by electron microscopy, i.e., direct observation of mitochondria in our results or light microscopy, i.e., indirect observation of mitochondria without observing any mitochondria directly and misinterpretation by Korr et al.[67,68,69,70]. Anyway, the increase and decrease of the intramitochondrial DNA synthesis may be related to the cellular function of the adrenal cortical cells in the three layers, which synthesized intramitochondrial DNA independently from the nuclear DNA synthesis and the mitochondria proliferated in respective cells due to aging. Thus, it is supposed that the secretions of the adrenal cortical hormones may depend on the mitochondrial energy production in relation to the intramitochondrial DNA synthesis in each adrenal cortical cell.

The results obtained from the adrenal glands of aging mice at present should form a part of special cytochemistry[48], as well as a part of special radioautographology[49], i.e., the application of radioautography to the adrenal glands, as was recently reviewed by the present author. We expect that such special radioautographology and special cytochemistry should be further developed in all the organs in the future.

### CONCLUSIONS

From the results obtained at present, it was concluded that almost all the cells in the three layers of the adrenal cortex of mice at various ages, from prenatal embryo day 19 to postnatal newborn, days 1, 3, 9, and 14, and to postnatal months 1, 2, and 6, were labeled with silver grains showing DNA synthesis with $^{3}$H-thymidine in their mitochondria. Quantitative analysis on the number of mitochondria in adrenal cortical cells in the three layers resulted in an increase from the prenatal day to postnatal days 1, 3, 9, 14, and months 1, 2, and 6, reaching the maximum at postnatal month 6. To the contrary, the numbers of labeled mitochondria with $^{3}$H-thymidine showing DNA synthesis and the labeling indices also increased from prenatal day 14 to postnatal day 14, and months 1 and 2, reaching the maximum at postnatal month 2, and then decreased to month 6. These results demonstrate that the number of mitochondria in adrenal cortical cells increased from perinatal stages to postnatal month 6 due to aging of animals, while the activity of mitochondrial DNA synthesis increased to month 2 at adult stage due to aging and decreased to month 6 due to senescence.

### ACKNOWLEDGMENTS

This study was supported in part by Grant-in-Aids for Scientific Research from the Ministry of Education, Science and Culture of Japan (No. 02454564) while the author worked at Shinshu University School of Medicine, as well as Grants for Promotion of Characteristic Research and Education from the Japan Foundation for Promotion of Private Schools (1997, 1998, 1999, 2000) while the author worked at Nagano Women's Jr. College. The author is also grateful to Grant-in-Aids for Scientific Research from the Japan Society for Promotion of Sciences (No. 18924034, No. 19924204, and No. 20929003) while the author worked at Shinshu Institute of Alternative Medicine from 2005 up to the present time. The author thanks Dr. Kiyokazu Kametani, Technical Official, Research Center for Instrumental Analysis, Shinshu University, for his technical assistance during the course of this study.
